# Collaborations in medical genetics: 10‐Year history of an ongoing Vietnamese‐North American Collaboration

**DOI:** 10.1002/mgg3.383

**Published:** 2018-04-16

**Authors:** Kathleen A. Leppig

**Affiliations:** ^1^ Genetic Services Kaiser Permanente of Washington Seattle WA USA; ^2^ Department of Pathology University of Washington Seattle WA USA

In 2006, one of my Group Health Cooperative (GHC) (now Kaiser Permanente of Washington) colleagues approached me with an opportunity to go on a medical exchange to Vietnam. Our institution had a more than 30‐year‐long collaboration, educational program, and physician exchange program focused on primary care medicine with Hue College of Medicine and Pharmacy in Vietnam. During his recent trip, my colleague met a medical geneticist, Dr. Nguyen Viet Nhan, who was working in Hue and seeking collaboration with a United States (US) based medical geneticist. This led to my first trip to Vietnam in 2007, which has been followed by nine subsequent trips and has been the basis of an ongoing and evolving collaboration between medical geneticists in Vietnam and medical geneticists, genetic counselors, and basic scientists in the United States and Canada. Our shared commitment is to provide educational support, genetic counseling, and clinical and molecular diagnostic expertise. This work has very importantly supported the development of genetic resources for patients in Vietnam and the physicians who provide this care, but it has also grown to involve the genetics communities in other Asia‐Pacific countries.

## 1 EDUCATION AND TEACHING

The initial work in Vietnam was focused on education, teaching medical genetics to medical students, nurses, pediatric physicians and pediatric specialists at Hue College of Medicine and Pharmacy. Lectures initially written in English were translated into Vietnamese, shown on dual screens and presented in both languages alternating between myself and my colleague from Vietnam. Although much of the medical education in Vietnam is given in English, providing the lectures in both languages was helpful because of the newness of genetics and the complexity of the terminology. These lectures have been used as an ongoing resource for Hue College of Medicine and Pharmacy as well as other medical schools in Vietnam.

In addition to the didactic lectures for medical students, other lectures have been provided on topics that include genomic testing for patients with a suspected underlying genetic condition, cancer genetics, genetic counseling, and the role of medical genetics in pediatric health care. In 2013, this collaboration resulted in the first Vietnamese‐North American Genetics Conference. This two day conference in Hanoi, Vietnam provided a forum for all the physicians, researchers, and other clinicians across Vietnam who deliver medical care to patients with suspected genetic conditions to meet and exchange information and resources with each other, and with experts from North America. The second Vietnamese‐North American Genetics Conference is being planned for 2018.

We partnered with the Asia‐Pacific Conference of Human Genetics in 2016 to develop a Genetic Counseling Preconference Workshop that was attended by over 90 individuals who provide genetic counseling across the Asia‐Pacific region. This workshop led to the development of the Professional Society of Genetic Counselors in Asia (Laurino et al., 2017, 2018). Attendance doubled to approximately 180 for the 2nd Genetic Counseling Preconference Workshop that occurred on November 8, 2017 in association with the 12th Asia‐Pacific Conference of Human Genetics in Bangkok, Thailand. These preconferences have been essential for genetic counselors and their allied healthcare partners in the Asia‐Pacific region to meet, develop collaborations, share resources, advocate for those who currently provide genetic counseling, and serve as a springboard for the development of new genetic counseling training programs. The importance of this work is validated by the plan to organize a 3rd Genetic Counseling Preconference Workshop in 2019.

A 2‐year masters program in genetic counseling is not currently available in Vietnam. There have been numerous requests to myself and my North American colleagues to develop genetic counseling short courses that would last several days to a week. However, this limited genetics education training cannot replace the robust training from a 2‐year masters program in genetic counseling. Plans have been recently announced to develop a genetic counseling program through Hanoi University with classes beginning fall of 2019. Learning from the experience of existing training programs in the region, we are supporting the medical geneticist in Vietnam with curriculum development (Laurino & Padilla, 2013).

Many of us have been gifted with excellent educations and opportunities to teach on varying academic levels in the United States and Canada. Providing access to similar education is frequently the first step to collaboration with health care providers in emerging countries who are in need of information, support and tools for the development of new technologies.

## 2 CLINICAL CARE

Training in medical genetics, and particularly dysmorphology, is more of an apprenticeship than fulfillment of a specific course requirement. The essential publications and tools to train in and practice dysmorphology, such as *Smith's Recognizable Patterns of Human Malformations* by Ken Lyon Jones (2013), have traditionally described syndromes based on findings of a western European population. The definitions of minor anomalies and normal variants that are used to characterize subtle physical findings in specific syndromes have only occurred for a Caucasian population (Leppig, Werler, Cann, Cook, & Holmes, 1987). Although there are growing attempts to include more photographs from affected individuals of different ethnic backgrounds in publications, there is not the same level of phenotypic detail provided for nonwestern European populations. There are several textbooks of dysmorphology specific for the population in Thailand (Shotelersuk, 2003), Peru (De Zighelboim, Jugo, Pastor, & Barriga, 2012), Japan (Kajii, Kuroki, Niikawia, & Fukushima, 1998) and South Africa (Windship, 2003), but none for many other regions of the world.

Currently, there are approximately 13 trained medical geneticists in Vietnam to care for nearly 90 million people. Given the volume of patients and lack of specific resources for the Vietnamese population, many of the patients who are seen in clinic with congenital malformations, growth abnormalities, and developmental disabilities remain undiagnosed. My colleagues and I have participated in diagnostic evaluations of patients in Vietnam during each one of our trips. Our goals have been the same as those when staffing clinics in North America: to help establish a correct diagnosis for the patient to direct clinical care and provide the family with recurrence risk information.

In addition to providing clinical expertise, we have tried to incorporate more cost effective and efficient diagnostics technologies than are currently available to diagnose genetic conditions in Vietnam. There are cytogenetics and molecular laboratories available in the major academic centers in Vietnam and specific molecular tests such as Fragile X testing have been developed. However, DNA microarray, whole‐exome sequencing (WES) and whole genome sequencing (WGS) are not routinely available for clinical care. In 2011, we collaborated with BlueGnome to use DNA obtained from 20 patients with suspected underlying genetic disorders (UGD) that had been evaluated in Genetics Clinic at Hue College of Medicine and Pharmacy using spit samples and blood samples for DNA microarray testing. The results of this pilot showed spit samples were inadequate in two patients. Of the 18 remaining patients, four (22%) had clear chromosomal imbalance and 4 (22%) patients had variants of unknown significance (unpublished data) (Table 1).

**Table 1:** Undiagnosed genetic disorders in Vietnam

Technique	DNA microarray	Whole exome sequencing
Year	2009	2016‐ongoing
Location	Hue	Hanoi
Total number of families	20	23
Inadequate sample	2	2
Other identified cause		1^a^
Pathogenic variants	4	16
Diagnostic yield	22% (4/18)	80% (16/20)

a Positive CMV titers in a child with microcephaly.

The diagnostic yield for identifying the etiology of a child suspected of having an UGD is approximately 15% by DNA microarray (Miller et al., 2010) and 25%–43% by WES (Monies et al., 2017; Wright et al., 2015; Yang et al., 2014). The ability to test pediatric patients in Vietnam with a suspected UGD has been enhanced by the collaboration with Dr. Mary‐Claire King's laboratory at the University of Washington. Thus far, 23 families have been evaluated for testing by WES, 13 patients were evaluated by myself and Jennifer Thompson in collaboration with physicians in Hanoi and 10 were submitted independently by Vietnamese physicians with a complete medical history, family history, physical examination, and photographs of the patients. When I was present during the evaluation, we were able to establish a likely diagnosis or differential diagnosis based on clinical history and physical examination for 70% of the cases. There were limitations for ascertaining a likely diagnosis when I was not present for the evaluation.

For these 23 families, DNA was extracted in Vietnam from the proband and, when possible, the parents and siblings (both affected and unaffected) and then sent to the King Laboratory at the University of Washington for sequencing, results analysis and possible candidate gene identification. Of the 23 patients studied thus far, one patient with negative sequencing results had microcephaly and positive titers for CMV; two other patients had inadequate DNA samples. Of the remaining 20 patients and/or families, a likely or possible etiology candidate gene has been identified in 16/20 (80%) of the patients and/or families (manuscript in preparation). The high diagnostic yield is likely the result of the severity of the phenotypes found in the probands and the availability of sequencing of multiple family members (Table 1).

The genetic conditions identified in this first group of patients include patients with Cockayne Syndrome, Robinow syndrome, Menkes syndrome, and Williams syndrome, and will help extend understanding of the phenotypic spectrum of patients with these conditions in a SE Asian population and serve as a basis of a Southeast Asian version of *Smith's Recognizable Patterns of Human Malformation*. Novel genetic conditions were identified including a child homozygous for a LZRT1 pathogenic variant, now recognized as an autosomal recessive form of Noonan syndrome, and was included in the manuscript describing an international cohort of patients (Johnston et al., 2018).

Vietnam has a national health care system where basic health care is available to the whole population. There is prenatal testing that is available when a specific pathogenic variant has been established for a patient. However, prenatal testing is only available in the private sector, with limited accessibility and high cost.

There are multiple clinicians from across the globe with varied expertise that visit medical institutions in Vietnam and other emerging countries and provide clinical care as individuals or a larger group as part of medical tourism. Even with regular annual visits to Vietnam, it is difficult to provide the consistency to move forward complex projects such as coordinating WES on patients with suspected genetic conditions. The challenges to support consistency are ongoing and require collaboration, creativity, persistence and commitment.

## 3 LIMITATIONS

One of the most important lessons learned from my work in Vietnam is to understand the different perception of time and the impact of limited resources. For example, at one point, a major communications cable was cut in Vietnam and I did not receive any communications from my Hanoi collaborators for several months. Not knowing the communication challenge, I was concerned. When there was a large measles outbreak in Vietnam in 2015, it forced the cancellation of an international genetics meeting as none of the physicians could take time away from urgently needed patient care to plan the conference. Priorities can quickly change when more urgent medical and social needs arise without our awareness.

The dedication and motivation of providers and families we have encountered to seek a diagnosis is apparent. We have been asked to perform diagnostic evaluations in remote schools for children with special needs without knowing in advance anything about the patients we will be evaluating. In these circumstances, there are unavoidable limitations for preparing for an appointment, ensuring all relevant family members attend, obtaining relevant clinical and family history, coordinating sample collection, and planning follow‐up.

While having the ability to extract DNA in Vietnam has allowed greater sample stability for shipping, collecting and transporting specific samples from patients continues to be challenging. RNA is not routinely extracted by the hospital laboratories in Vietnam and shipment of biologic samples such as blood or skin biopsies are under strict regulation and can be delayed in customs. Clinical evaluations such as a cranial imaging, echocardiograms, ophthalmologic examinations, etc., can be extremely difficult to obtain because of the limitation of machinery and trained personnel to perform testing and interpret results. These limitations can result in impediments for obtaining full clinical and molecular evaluation, and delay the diagnostic process. Patience and persistence are necessary to coordinate sample collection and complete studies when working with physicians, their patients, and families who are thousands of miles away. While barriers can be frustrating, there is always another way to move the work forward, and moving forward is what is most essential to this work.

## 4 FUNDING

While the role of educator, clinician, and organizer are easy to assume, there are other roles that are necessary for the work in Vietnam which have been more difficult and lie beyond medical school training. Chief among these has been fundraiser. The average physician in Vietnam earns approximately $1,200 USD per year! When trying to develop a conference such as the first Vietnamese‐North American Genetics Conference and the Genetic Counseling Pre‐conference Workshops for the Asia‐Pacific Conference of Human Genetics, fundraising was necessary to secure monies to pay for the venue, meals, and support travel and accommodations for invited speakers. Securing funding for projects such as this is extremely difficult. There are currently no National Institutes of Health grants for supporting international meetings of this nature. There are more than 1,000 non‐government organizations (NGO's) in Vietnam, but none we've identified with a specific mission of medical genetics. The greatest success in securing funding came from companies that perform genetic testing. But even with securing funds, it is important to manage funds in a tax‐structure as intended for nonprofit work. Group Health Foundation initially provided this financial structure, but ultimately, forming a NGO becomes unavoidable to manage funds efficiently and pursue a consistent program to support medical genetics in Vietnam. We are in the process of developing such an NGO, identifying board members and defining bylaws to meet the intended goals and expectations of this work.

## 5 FUTURE DIRECTIONS

For all, of my trips to Vietnam, I still have only a handful of Vietnamese words that I can speak, and not many more that I understand. Tonal languages such as Vietnamese plus the issue of multiple dialects in this region make it particularly difficult to acquire useful language and communicate. I know that I miss many of the nuances in conversations between patients and the Vietnamese physicians. As an outsider, it would be difficult to perform any type of independent health care in Vietnam and one hopes to support the develop of resources and transfer of technology that has been done successfully before in other regions of the world, particularly the Mediterranean (Özçelik et al., 2010). Ongoing collaborations that include the next Vietnamese‐North American Conference in November, 2018, ongoing collaborations with Mary‐Claire King's laboratory, supporting the development of a genetic counseling program and spending time in clinic in Hanoi, Hue, and potential other genetics clinic in Vietnam will all facilitate the growth of genetic services and genetic and genomic expertise within Vietnam.

## 6 WHY VIETNAM?

One of the first questions I am frequently asked when describing my work is “Why Vietnam?” Part of the answer is easy: GHC had a long‐time collaboration with Hue College of Medicine and Pharmacy for a medical exchange to train primary care practitioners. I was surprised and very excited about the opportunity when my GHC colleagues returned from Vietnam, saying that physicians in Hue wanted to established collaborations with a medical geneticist.

The second part of the answer is much more difficult. If you were born in the United States (US) in the 1940 and 1950s, it would be difficult not to be aware of the War in Vietnam (1955–1975) and the ramification of this war in both the United States and Vietnam. Three of my male cousins and my husband were all of age during the Vietnam War and all were impacted by the war in different ways. One cousin had a very low draft number but was ultimately excused from military service as he was in graduate school for metallurgy and deemed to be too valuable to the United States to be sent to war. My second cousin graduated from West Point Academy, subsequently joined the Marine Corps, and flew multiple missions over Vietnam only to die in a training accident off the Marine Corp Air Station in Cherry Point, North Carolina. My third cousin was drafted out of high school, fought with the ground troops in Vietnam, returned back to the United States after his year of service addicted to drugs, and was killed in a motorcycle accident when he was in the process of recovering from his addiction and beginning his college education. My husband was excused from military service as he was enrolled in medical school; that exclusion criteria changed later in the war.

The impact of the war in both the United States and Vietnam was different, but weighty for both countries. Over three million Vietnamese lost their lives during the war with destruction of infrastructure and defoliation of approximately 10% of the land. I started to work in Vietnam simply to try to do something that would contribute to the betterment of people of Vietnam, using my training in medical genetics. While there is no financial compensation for this type of volunteer work, the rewards have been innumerable. The physicians in Vietnam whom I have encountered are among the kindest and hardest working people I have ever met. Working with them in clinic and helping support the work to provide patients with a diagnosis and care has been among the most valuable experiences of my professional life. I am looking forward to being able to spend a longer stretches of time in Vietnam during a planned sabbatical and when I retire from clinical practice and hope to remain involved in the growth of medical genetics in Vietnam with my co‐collaborators for years to come.

## 7 THANKS

The work in Vietnam would not have been possible without the support of many people including my North American colleagues Mercy Laurino, Jennifer Thompson, and Darci Sternen, all genetic counselors who have been essential to this work, Mary‐Claire King and Suleyman Gulsunar whose molecular expertise and generosity are extraordinary, and my colleagues in Vietnam, particularly Nguyen Viet Nhan and Du Chi Dung whose dedication to helping those with genetic disorders is an inspiration to us all. And finally to my family, thank you for supporting me and joining me in what has become one of the defining experience of my professional life.

## CONFLICTS OF INTEREST

None declared.
